# Polyunsaturated fatty acids in lipid membranes regulate human neuronal function and amyloid-β production

**DOI:** 10.1016/j.isci.2025.112557

**Published:** 2025-05-12

**Authors:** Satoshi Morita, Takayuki Kondo, Hisanori Tokuda, Yoshihisa Kaneda, Takayuki Izumo, Yoshihiro Nakao, Haruhisa Inoue

**Affiliations:** 1 iPSC-based Drug Discovery and Development Team, RIKEN BioResource Research Center (BRC), Kyoto, Japan; 2 Institute for Science of Life, Suntory Wellness Ltd., Kyoto, Japan; 3 Center for iPS Cell Research and Application (CiRA), Kyoto University, Kyoto, Japan; 4 Medical-risk Avoidance based on iPS Cells Team, RIKEN Center for Advanced Intelligence Project (AIP), Kyoto, Japan

**Keywords:** Neuroscience, Cell Biology, Molecular Biology

## Abstract

The effects and mechanisms of polyunsaturated fatty acids (PUFAs) including docosahexaenoic acid (DHA) and arachidonic acid (ARA) contained in the lipid membrane of neurons in the production of amyloid β (Aβ), a pathogenic molecule in Alzheimer’s disease (AD), remain unclear. In this study, we cultured human cortical neurons differentiated from induced pluripotent stem cells (iPSCs) under conditions of PUFA deficiency being progressively alleviated. Under PUFA-deficient conditions, increasing the total PUFA composition ratio in the lipid membrane enhanced membrane fluidity and reduced Aβ production. Furthermore, in conditions where the overall PUFA deficiency was resolved, altering the specific ratios of DHA and ARA promoted the synchronous activity and morphological complexity of neuronal cells while maintaining consistent membrane fluidity. These findings demonstrate that the overall PUFA composition in the lipid membrane as well as the specific ratios of DHA and ARA within the total PUFAs regulate neuronal function and pathophysiology.

## Highlights

• PUFAs in lipid membranes regulates membrane fluidity in human neuronal cells
• Enhanced membrane fluidity decreases the production of Aβ
• Enhanced composition of DHA and ARA increases neuronal complexity and synaptic growth
• Enhanced composition of DHA and ARA increases synchronized neuronal activity

## Graphical abstract

**Figure:**
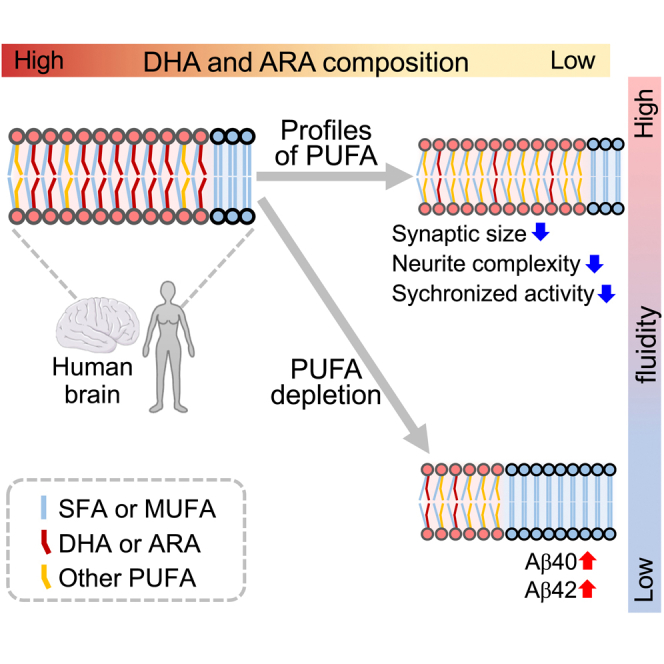

## Introduction

Consuming nutrients from our daily diets is crucial for proper brain function.^1^ Among these nutrients, lipids are particularly noteworthy as they make up over half of the brain’s dry weight, mostly in the form of phospholipids.^2^^,^^3^^,^^4^ Polyunsaturated fatty acids (PUFAs), including docosahexaenoic acid (DHA) and arachidonic acid (ARA), are especially abundant in the brain and are obtained via our daily diet.^5^^,^^6^^,^^7^ Research has shown that consuming PUFAs can have beneficial impacts on brain function in humans,^8^^,^^9^^,^^10^^,^^11^ although a deficiency in these nutrients has been associated with an elevated risk of diseases.^12^ However, the exact role of PUFAs in neurological functions and pathological conditions is still not fully understood.

Lipid membranes have a significant impact on cellular functions.^13^ Alterations in lipid membranes within the brain have been linked to aging and neurodegenerative diseases. Regarding the fatty acid compositions of lipid membranes in the brain, the levels of DHA and ARA are known to decline with age.^5^^,^^14^^,^^15^ Additionally, individuals with Alzheimer’s disease (AD) have presented reduced levels of DHA and ARA in the brain.^14^^,^^16^ Furthermore, the physical properties of lipid membranes are not constant, as there are areas of high fluidity and of low fluidity, and increased PUFA in lipid membranes is known to enhance lipid membrane fluidity.^17^^,^^18^^,^^19^ The fluidity of lipid membranes has an impact on the distribution of proteins in membranes, including enzymes that cleave amyloid precursor protein (APP). APP is a precursor protein of amyloid beta (Aβ), a neuropathological hallmark of AD.^20^ The association between Aβ accumulation and AD pathogenesis has been thoroughly studied since the amyloid cascade hypothesis was proposed. APP undergoes sequential proteolytic processing via two distinct pathways: the non-amyloidogenic pathway mediated by α-secretase, and the amyloidogenic pathway involving β- and γ-secretases. In the amyloidogenic pathway, APP is first cleaved by β-secretase, followed by γ-secretase-mediated cleavage. The more hydrophobic Aβ42 is more prone to aggregation and to form oligomers, which are considered the primary toxic species in AD pathogenesis. The fluidity of lipid membranes could alter the APP cleavage process.^21^^,^^22^^,^^23^^,^^24^

Neural cells differentiated from human induced pluripotent stem cells (iPSCs) are an appropriate model for reproducing the human brain in a culture dish.^25^ However, this has not been sufficiently considered for the reproduction of the characteristic lipid membrane state of the human brain. Brain lipid composition is different from that of non-neural tissues.^6^ In particular, human brain has been shown to differ from mouse brain in terms of lipid characteristics.^26^ In this study, we utilized an *in vitro* model that differentiated neurons from human iPSCs. We then modified the fatty acid compositions in lipid membranes to investigate how changes in lipid membranes affect neuronal function and pathophysiology. We established a model where the overall PUFA composition ratio and membrane fluidity were significantly altered, and also a model where the type of PUFA was recomposed while maintaining the overall PUFA composition and fluidity. In both conditions, we observed that the *in vitro* models’ lipid membrane characteristics closely rescapitulated those found in the human brain.^6^ Our findings highlight the significance of lipid traits of the human brain in terms of both the quantitative and qualitative perspectives of PUFA.

## Results

### Enhanced PUFA composition in phospholipids reduced Aβ40 and Aβ42 production

**Figure 1:**
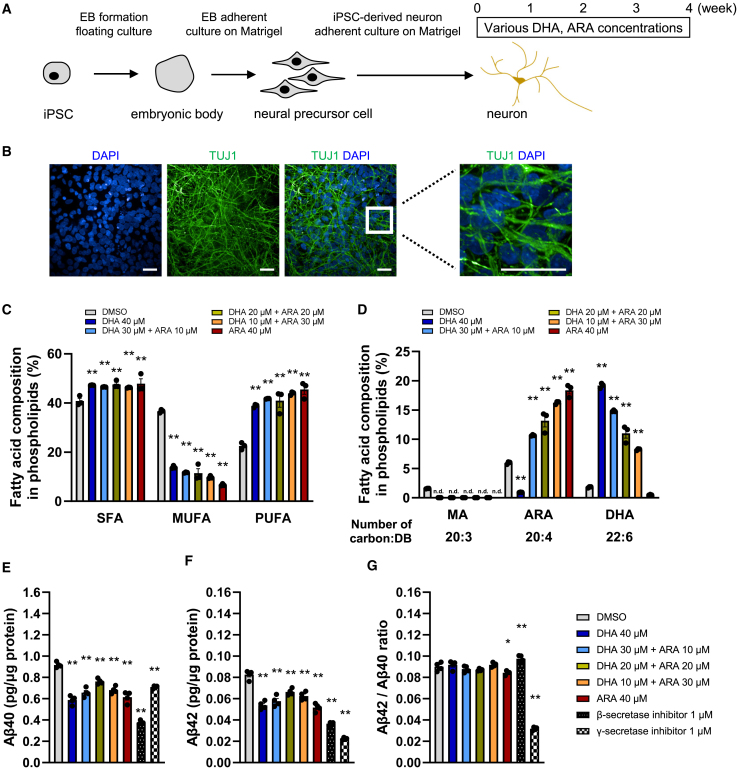
Enhanced PUFA composition in phospholipids reduced Aβ40 and Aβ42 production (A) Schematic diagram of conditions used to induce iPSC-derived neurons. (B) Representative images of iPSC-derived neurons at 4 weeks. Scale bars, 25 μm. (C) Total compositions of saturated, monounsaturated, and PUFAs in phospholipids in cells cultured for 4 weeks with DHA and/or ARA. Data represent mean ± SE, *n* = 3, Dunnett’s test compared to DMSO control, ∗*p* < 0.05 and ∗∗*p* < 0.01. (D) Compositions of MA, ARA, and DHA in phospholipids in cells cultured for 4 weeks with DHA and/or ARA. Data represent mean ± SE, *n* = 3, Dunnett’s test compared to DMSO control, ∗∗*p* < 0.01. n.d., data not detected. See also Tables S1 and S2. (E–G) The amount of Aβ40 (E), Aβ42 (F), and the ratio of Aβ42/Aβ40 (G) in a 48 h cultured medium was analyzed. DHA and/or ARA were treated for 4 weeks, whereas β-secretase inhibitor and γ-secretase inhibitor were treated for only the last 48 h. Data represent mean ± SE, *n* = 4. Dunnett’s test compared to DMSO control, ∗*p* < 0.05 and ∗∗*p* < 0.01.

First, we created a model in which the PUFA proportion and membrane fluidity were altered by utilizing a medium with linoleic acid (LA) and α-linolenic acid (ALA) as exclusive PUFA sources. We used iPSCs derived from healthy individuals. DHA and/or ARA were treated on differentiated neurons for 4 weeks (Figure 1A). The expression of neuronal marker proteins was confirmed by immunostaining (Figure 1B). The culture system contained LA and ALA as PUFA in the culture medium, while DHA and ARA were not detected (Table S1). DHA and/or ARA were treated as achieving the same total concentration. We confirmed that DHA and/or ARA addition for 4 weeks did not affect neuronal differentiation (Figure S1). The total amount of monounsaturated fatty acids (MUFAs) decreased by approximately 30%, while the total amount of PUFA increased about 1.8-fold (Figure 1C). The total amount of saturated fatty acids (SFAs) increased slightly to about 1.2-fold. No differences were observed in the total amounts of SFA, MUFA, and PUFA when DHA or ARA were added alone, or when they were added in combination. The amounts of DHA and ARA in the phospholipids reflected the amounts of DHA and ARA added to the medium, and other fatty acids in phospholipids greatly adapted (Figure 1D; Table S2). Mead acid (MA), a fatty acid that is alternatively biosynthesized in the PUFA-deficient condition,^27^^,^^28^ was detected in cells without PUFA, but the addition of DHA and/or ARA eliminated MA. This means that the PUFA deficiency was resolved by the addition of DHA and/or ARA in this model. Since alteration of the lipid membrane environment is associated with the Aβ cleavage process on the lipid membrane,^21^^,^^22^^,^^23^ we measured the amount of Aβ in the culture supernatants. In the PUFA-deficient condition, enhanced PUFA composition reduced Aβ40 (Figure 1E) as well as Aβ42 (Figure 1F). Aβ40 and Aβ42 were similarly reduced, with no significant alterations in the Aβ42/Aβ40 ratio (Figure 1G). The effects of enhanced PUFA composition were similar to those of the β-secretase inhibitor. No major differences were observed between the addition of either DHA or ARA, or with their combination, suggesting the importance of the enhanced overall PUFA composition rather than the specific types of PUFA.

### Enhanced PUFA composition in phospholipids increased lipid membrane fluidity

**Figure 2:**
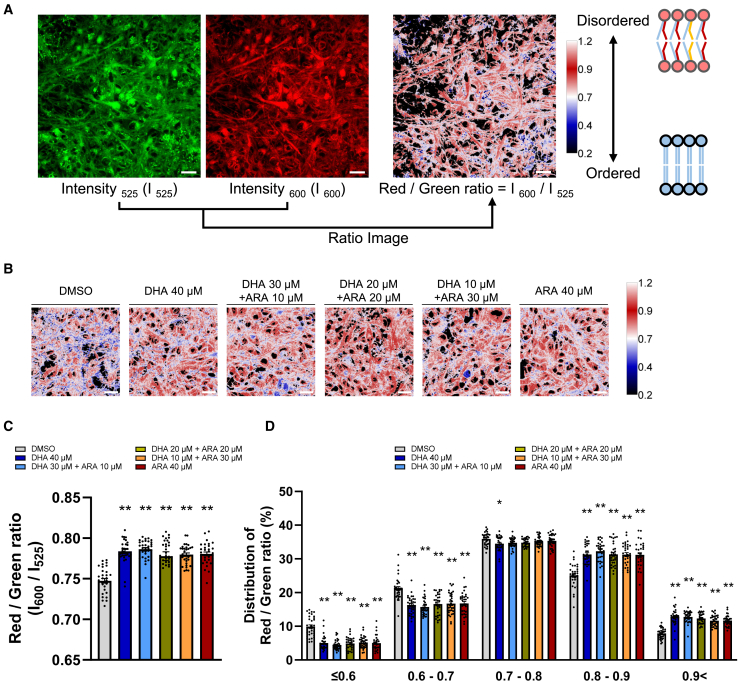
Enhanced PUFA composition in phospholipids increased lipid membrane fluidity (A) Lipid membrane fluidity was analyzed using a fluorescent probe, characterized by a fluorescence wavelength transition from 525 nm (green) to 600 nm (red), depending on the increasing fluidity of the lipid membrane. The calculated red/green ratio is shown in pseudocolor. Scale bars, 20 μm. (B) Representative images of the red/green ratio after being treated with DHA and/or ARA for 4 weeks. Scale bars, 20 μm. (C) The red/green ratio was calculated for each picture. Data represent mean ± SE, 30 images were analyzed from 3 independent biological samples for each condition. Kruskal-Wallis test followed by Dunn’s test compared to DMSO control, ∗*p* < 0.05 and ∗∗*p* < 0.01. (D) Distribution of red/green ratio for each image field. Data represent mean ± SE, 30 images were analyzed from 3 independent biological samples for each condition. Kruskal-Wallis test followed by Dunn’s test compared to DMSO control, ∗*p* < 0.05 and ∗∗*p* < 0.01.

Aβ production occurs on the lipid membranes of neurons, and PUFA has been reported to increase the fluidity of lipid membranes.^17^^,^^18^^,^^19^ We investigated how the properties of lipid membranes change under the PUFA altered condition. Lipid membranes could be directly monitored using a fluorescence probe (LipiORDER) to determine the lipid order in live cells.^29^ The LipiORDER probe shows strong solvatochromism and an emission color response to the lipid order in membranes (ordered vs. disordered liquid phases). Lipid ordered and disordered phases can be differentiated by a shift in fluorescence wavelength according to the membrane phases. Those lipid membrane phases are involved in the formation of lipid microdomains (rafts) in cell membranes.^30^ We evaluated the order-disorder state of membranes using LipiORDER, and the ratio of fluorescence intensity 600/525 nm was calculated (Figure 2A). DHA and/or ARA increased the ratio of fluorescence intensity 600/525 nm (Figures 2B and 2C). The histogram also showed an overall shift toward a higher ratio, indicating that the ordered regions decreased as the disordered regions increased in the cellular lipid membrane (Figure 2D). Since it is well known that Aβ is produced by the cleavage process of APP on neuronal endosome membranes,^31^ the ratio image of 600 nm/525 nm was overlaid with endosomal regions. We did not observe significant differences between the membrane fluidity measurement of overall cellular membranes combined and those of endosomal subsets (Figure S2). These results suggest that endosomal membrane in this culture system may also be reconstituted by lipid supplementation. Cholesterol is a well-known representative factor that decreases the fluidity of lipid membranes.^32^^,^^33^ We investigated whether the alterations of membrane fluidity and Aβ production observed with DHA and ARA could also be observed with cholesterol. The addition of DHA and/or ARA did not affect the cellular cholesterol level (Figure S3A). Methyl-β-cyclodextrin (MβCD), which pulls cholesterol from lipid membranes, decreased the amount of cholesterol in a concentration-dependent manner (Figure S3B). MβCD increased membrane fluidity (Figure S3C), and Aβ40 and Aβ42 productions were both decreased (Figures S3D and S3E). The Aβ42/Aβ40 ratio was decreased, unlike the results from DHA and/or ARA (Figure S3F). Therefore, membrane fluidity alterations affected the APP cleavage process and decreased Aβ40 and Aβ42 production.

### Neuron-astrocyte coculture system

**Figure 3:**
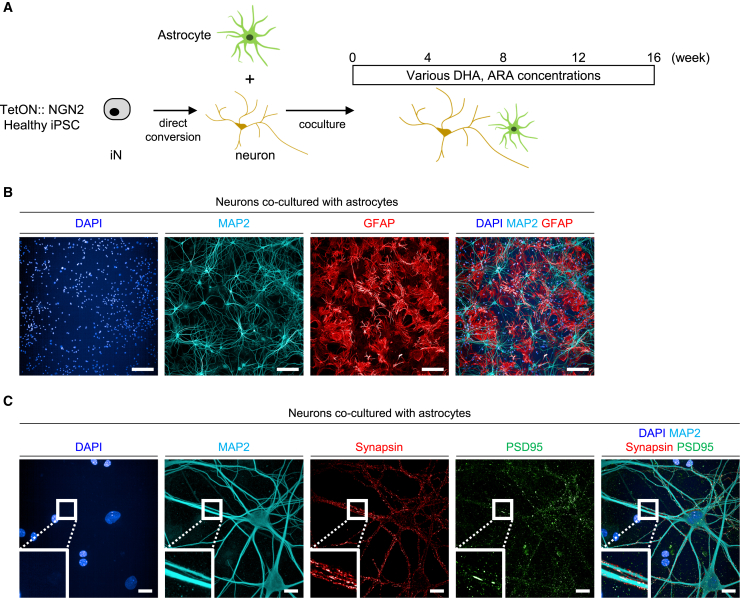
Neuron-astrocyte coculture system (A) Schematic diagram of coculture conditions. Neurons and astrocytes were mixed on day 5, and then DHA and/or ARA were treated for 16 weeks. (B) Representative images of neurons and astrocytes at week 16. Scale bars, 200 μm. (C) Representative image of pre-/post-synaptic sites of neurons at week 16. Scale bars, 20 μm.

Next, we established another model in which the profiles of PUFA were recomposed while maintaining the overall PUFA composition and membrane fluidity under PUFA-sufficient conditions. Since long-term culture is required to evaluate functional activities in human iPSC-derived neurons,^34^ and astrocytes play an important role in lipid metabolism in the brain,^35^^,^^36^ we used a coculture system in which healthy iPSC-derived neurons were combined with brain-mixed astrocytes (Figure 3A). The amount and profiles of PUFAs included in the culture medium completely differed from those in the PUFA-deficient model (Table S3). DHA and ARA, separately or in combination, were added to the medium at higher concentrations than those present in the culture medium. Human neurons were differentiated from healthy human iPSCs using direct conversion technology by inducing transient expression of NGN2 in iPSCs.^37^^,^^38^ Immunostaining confirmed the expression of neuron and astrocyte marker proteins, as well as the formation of pre-/post-synaptic sites on the neurites (Figures 3B and 3C), thereby indicating the formation of the functional neuronal network.

### DHA and ARA altered neurite outgrowth

**Figure 4:**
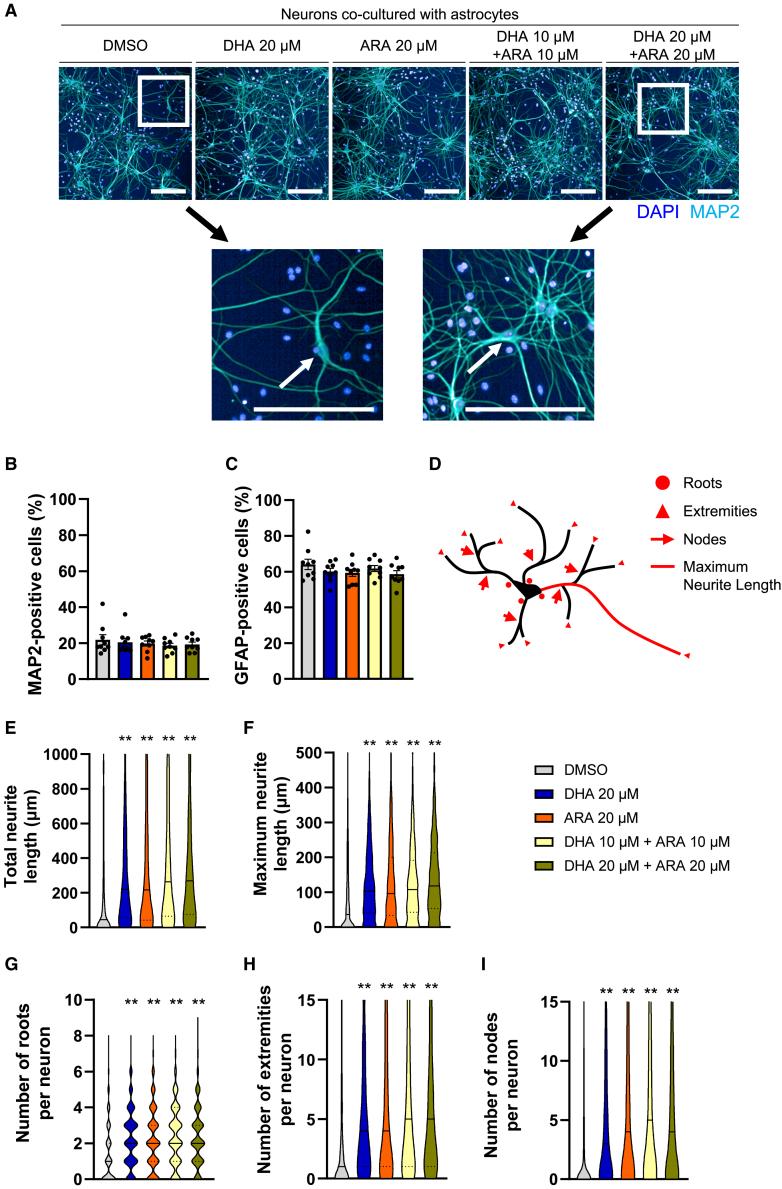
DHA and ARA promoted neurite outgrowth (A) Representative images of neurons cultured for 16 weeks with DHA or ARA alone, or together. White arrows indicate representative neurites in extended larger pictures. Scale bars, 200 μm. (B and C) Number of MAP2/GFAP-positive cells was calculated. Data represents mean ± SE of 9 fields for each condition. (D) Each parameter was defined to evaluate the characteristics of neurites. Circle: number of roots per cell body; angle: number of nodes on neurites per neuron; triangle: number of extremities per neuron; red line: longest neurite of each neuron. Total neurite length was also calculated as the sum of the neurite length of each neuron. (E–I) Each parameter was calculated from images of neurons. Data represents mean ± SE, *n* = 1,592; 1,060; 1,027; 1,096; and 865 neurons from 9 images for each condition. Kruskal-Wallis test followed by Dunn’s test compared to DMSO control, ∗∗*p* < 0.01.

We examined the characteristics of neuronal morphology as the associations of DHA, ARA and neuronal morphology were previously reported in epidemiological studies^39^^,^^40^ and in *in vitro* studies,^41^^,^^42^ but little was known regarding human neurons, and especially the effects of ARA and their combinations. Neurite characteristics were analyzed in immunostained neurons (Figure 4A). The ratio of neurons or astrocytes in the coculture system did not change after the addition of fatty acids for 16 weeks (Figures 4B and 4C). Long neurites and greater numbers of branches on neurites support synaptic connections,^43^ and they decrease in aged human brain.^44^^,^^45^^,^^46^ In order to quantify the neurite characteristics, the following parameters were analyzed: the number of neurites extending from each cell body (roots), the number of branches present on the neurites (nodes), the number of neurite endpoints (extremities), the maximum neurite length of each neuron, and the sum of neurite length of each neuron, respectively (Figures 4D and S4). DHA and/or ARA increased the total neurite length, maximum neurite length, the number of roots, extremities, and nodes (Figures 4E–4I). The effects were similar when the culture medium was supplemented with DHA or ARA alone or with their combination. These results suggested that DHA and/or ARA promoted the formation of complex neuronal morphology.

### DHA and ARA affected synapse formation

**Figure 5:**
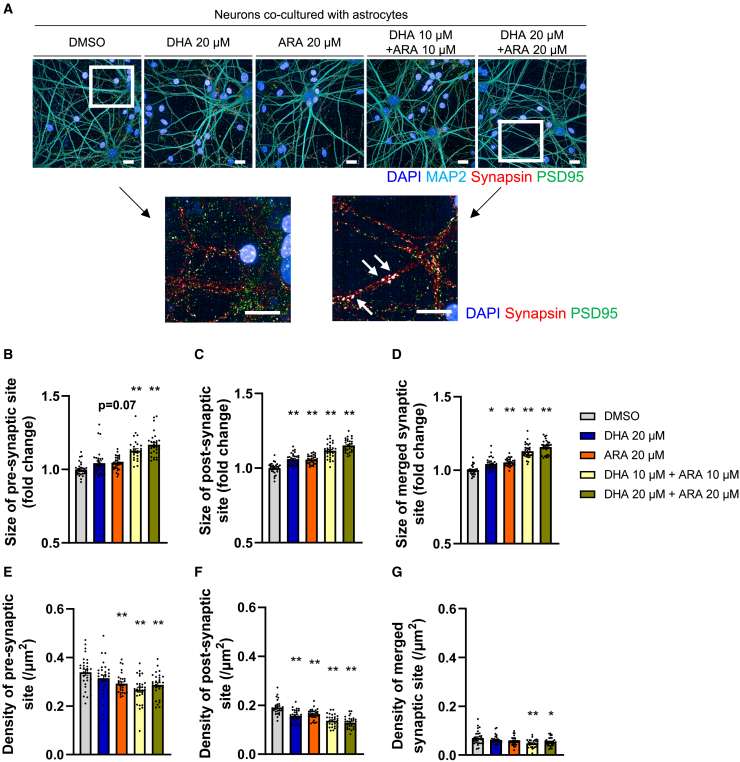
DHA and ARA affected synaptic formation (A) Representative images of neurons cultured with DHA or ARA alone, or together. White arrows indicate representative large synapses in extended large pictures. Scale bars, 20 μm. (B–D) Numbers of pre-synaptic sites, post-synaptic sites, and merged regions were counted and presented as density (/μm^2^). Data represent mean ± SE, 30 images were analyzed from 3 independent biological samples for each condition. Kruskal-Wallis test followed by Dunn’s test compared to DMSO control, ∗*p* < 0.05 and ∗∗*p* < 0.01. (E–G) Size of the pre-synaptic sites, post-synaptic sites, and merged regions was calculated. Data represent mean ± SE, 30 images were analyzed from 3 independent biological samples for each condition. Kruskal-Wallis test followed by Dunn’s test compared to DMSO control, ∗*p* < 0.05 and ∗∗*p* < 0.01.

To further investigate the effects of DHA and ARA on the neuronal morphology, their effects on synapse formation were analyzed using immunostained neurons. Regulation of synaptic densities and growth are related to the stabilization of synapses during synaptic plasticity.^47^^,^^48^ Pre-synaptic sites, post-synaptic sites, and merged regions were immunostained and their densities and sizes were analyzed (Figures 5A and S5). Synapsin was used as a pre-synaptic site marker and PSD-95 as a post-synaptic site marker. The sizes of the pre-synaptic sites, post-synaptic sites, and merged regions were both increased as shown in the representative extended images in Figure 5A. Image analysis revealed that the effects of either DHA or ARA alone on synaptic size were similar, and stronger effects were observed when they were combined (Figures 5B–5D). As synaptic size is one of the characteristics of synaptic maturation,^41^ either DHA or ARA, and even more when combined, could promote synaptic maturation. On the other hand, the densities of the presynaptic and postsynaptic sites were both unchanged by either DHA or ARA alone. However, when combined, the densities decreased (Figures 5E–5G). DHA and ARA had a significant impact on the size and density of synapses present on the neurons.

### DHA and ARA increased synchronized neuronal activity

**Figure 6:**
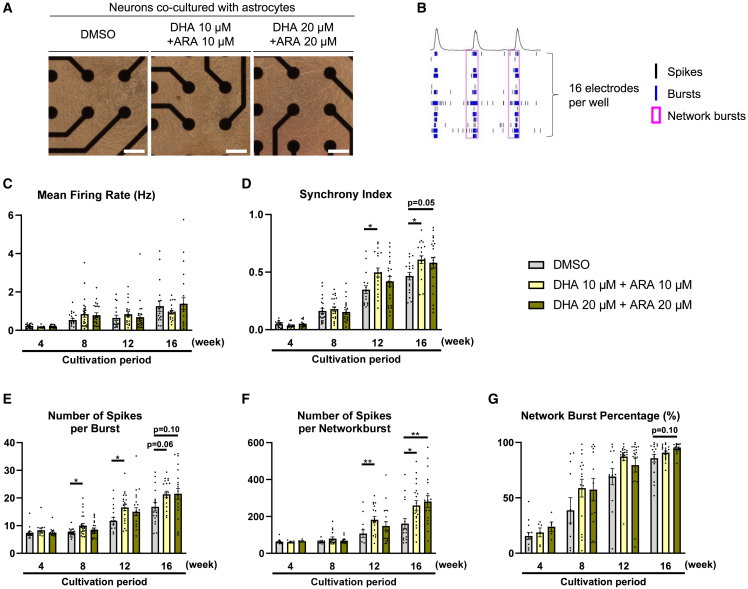
DHA and ARA increased synchronized neuronal activity (A) Representative light field images of cells cultured for 16 weeks. Neurons and astrocytes were cocultured on MEA plates and neuronal activity was measured over time, at 4, 8, 12, and 16 weeks. Scale bars, 200 μm. (B) Raw data of recorded activity and calculated parameters to evaluate the neuronal activity. Definitions of spikes, bursts, and network bursts are described in STAR Methods. (C–G) Mean firing rate (C) means the number of spikes per minute, which did not change significantly with DHA and ARA. The parameter representing the neuronal connectivity is shown in (D). Neuronal connectivity was increased by DHA and ARA. Similar parameters as the synchrony index are shown in (E– G), which represent simultaneous activity with other neurons in the same well. Data represents mean ± SE, *n* = 13–24, Kruskal-Wallis test followed by Dunn’s test compared to DMSO control, ∗*p* < 0.05 and ∗∗*p* < 0.01.

Since alterations of neuronal morphology and synapse formation might affect neuronal activity, we examined the effects on functional neuronal activity and connectivity. We examined the effects when DHA and ARA were combined, as the combination of DHA and ARA had potent effects on neuronal morphology. Neurons and astrocytes were cultured for 16 weeks on multi-electrode array (MEA) plates, and action potentials were recorded over time. Cells remained viable on the electrode surfaces throughout the 16-week experimental period (Figure 6A). We evaluated the characteristics of neuronal activities as follows: individual spike frequencies, burst frequencies, and interactions with other electrodes in the same well (Figure 6B). It was indicated that the spike frequency (mean firing rate) increased during the culture period, but the effects of DHA and ARA could not be confirmed (Figure 6C). On the other hand, the Synchrony Index, a parameter of activity synchrony, not only increased during the cultured periods but also increased by DHA and ARA (Figure 6D). Similar parameters representing the neuronal activity with the other neurons in the same well showed similar effects on the Synchrony Index, such as the number of spikes per burst (Figure 6E), the number of spikes per network burst (Figure 6F), and the network burst percentage (Figure 6G). These results therefore suggested that DHA and ARA improved neuronal connectivity.

### Enhanced DHA and ARA composition in phospholipids but stable membrane fluidity in the neuron-astrocyte coculture system

**Figure 7:**
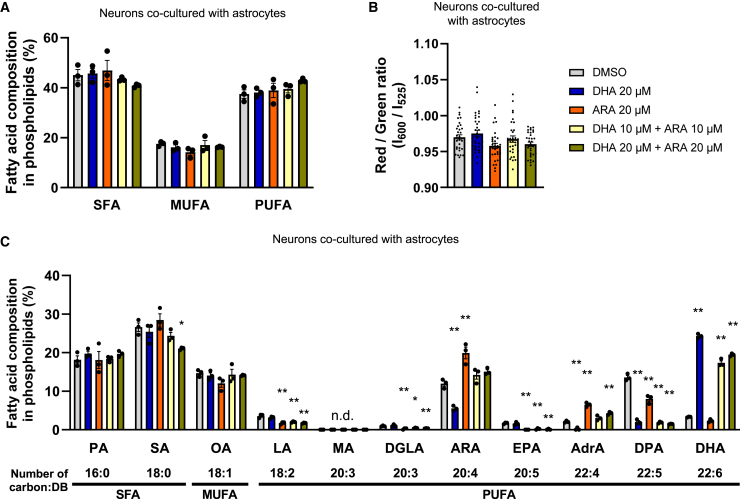
Enhanced DHA or ARA composition in phospholipids but stable PUFA composition and membrane fluidity (A) Fatty acid compositions of saturated fatty acids (SFA), monounsaturated fatty acids (MUFA), and polyunsaturated fatty acids (PUFA) in phospholipids are presented as mean ± SE, *n* = 3. (B) Lipid membrane fluidity was analyzed by LipiORDER fluorescent probe, and the red/green ratio was calculated. Data represent mean ± SE, 30 images were analyzed from 3 independent biological samples for each condition. (C) Compositions of each fatty acid in phospholipids in cells cultured for 16 weeks with DHA or ARA alone, or together. Data represents mean ± SE, *n* = 3; Dunnett’s test compared to DMSO control, ∗*p* < 0.05 and ∗∗*p* < 0.01. PA, palmitic acid; SA, stearic acid; OA, oleic acid; LA, linoleic acid; DGLA, dihomo gamma linolenic acid; ARA, arachidonic acid; EPA, eicosapentaenoic acid; AdrA, adrenic acid; DPA, docosapentaenoic acid; DHA, docosahexaenoic acid. See also Tables S3 and S4.

Under the PUFA-sufficient condition, no significant change in the total amounts of SFA, MUFA, or PUFA was observed with DHA or ARA alone, nor together (Figure 7A). Lipid membrane fluidity was not affected by DHA and/or ARA (Figure 7B). We did not observe significant differences between the membrane fluidity measurements of all cells combined and those of neurons alone, suggesting that neurons might be affected by lipid supplementation to a similar extent as astrocytes (Figures S6A and S6B). MA is a fatty acid that is alternatively biosynthesized in the PUFA-deficient condition.^27^^,^^28^ However, MA was not detected in the cells, which means that the amount of PUFA is sufficient in this coculture system (Figure 7C). We referenced and calculated the fatty acid compositions in the human brain from a previous study,^6^ and compared differences between the cocultured cells and the human brain (Figure 7C; Table S4). In this model, DHA, ARA, LA, ALA, dihomo gamma-linolenic acid (DGLA), eicosapentaenoic acid (EPA), n-3 docosapentaenoic acid (DPA), and adrenic acid (AdrA) were major detected PUFAs. When only one of DHA or ARA was added, the other was decreased. However, when both were added, both were incorporated in a balanced manner. On the other hand, the amounts of LA, DGLA, EPA, and n-3 DPA were significantly decreased as respective replacements. The alteration of AdrA composition was similar to that of ARA. Enhanced DHA and ARA composition recomposed the profiles of PUFA, while the total PUFA proportion and membrane fluidity remained stable. Therefore, in this model, overall PUFA was sufficient as a total proportion but enhanced the compositions of DHA and ARA. These results suggested that neuronal synchronized activities were enhanced even when the total PUFA proportion and membrane fluidity were not altered. We analyzed the Aβ levels under a condition of no changes in membrane fluidity. As a result, no significant changes were observed in Aβ40, Aβ42, or the Aβ42/Aβ40 ratio by the addition of DHA and ARA (Figures S7A–S7C). These results suggested that DHA and ARA might affect Aβ production through changes in membrane fluidity.

## Discussion

We established models to examine the effects of altering the ratio of PUFA composition and membrane fluidity, as well as recomposing the profiles of PUFA remaining stable of its overall PUFA proportion and membrane fluidity. Our findings revealed reducing Aβ levels when PUFA composition and membrane fluidity were increased. Even when the total PUFA proportion and membrane fluidity remained unchanged, the synchrony of neuronal activity improved by recomposing the profiles of PUFA.

### The composition of PUFA and the membrane fluidity effect on the processing of APP cleavage

In the PUFA-deficient condition, both the amounts of Aβ40 and Aβ42 decreased by approximately 0.6- to 0.8-fold when we increased the PUFA composition and lipid membrane fluidity. The effects on Aβ production were similar to the effect of β-secretase inhibitor. Previous studies have shown that changes in the lipid membrane environment affected APP processing.^21^^,^^22^^,^^23^^,^^24^ It was suggested that β-secretase localizes to the ordered lipid rafts region, while α-secretase localizes to the disordered non-lipid rafts region, and access of α-/β-secretase to APP is the key factor for regulating the Aβ cleavage process.^21^ The shift from ordered to disordered regions might increase the α-secretase-mediated cleavage process of APP more than that of β-secretase, resulting in lower amounts of Aβ40 and Aβ42. It has been reported that either DHA or ARA increases membrane fluidity in the brain,^17^ and the present report is the first to confirm this effect in human neurons.

We used the LipiORDER fluorescent probe to measure the fluidity of lipid membranes.^29^ This probe can pass through the plasma membrane and enter the intracellular lipid membrane, and it can exist mainly in lipid membranes that constitute various intracellular organelles such as mitochondria, endoplasmic reticulums, golgi, endosomes, and lysosomes, which are the major phospholipids that constitute the cells.^49^^,^^50^ It is well known that Aβ is produced by the cleavage process of APP on neuronal endosome membranes.^31^ No significant differences were observed between endosomal membrane fluidity and overall cellular membrane fluidity following fatty acid supplementation, suggesting that endosomal membrane reconstitution occurred after the addition of DHA and ARA. We modified the membrane fluidity not only by altering the composition of PUFA but also by altering the cholesterol amount, a typical regulator of membrane fluidity.^33^^,^^51^ By reducing the amount of cellular cholesterol, enhanced membrane fluidity in turn led to a decrease in Aβ40 and Aβ42. This finding was consistent with previous studies that demonstrated that cholesterol metabolism and transport play a role in regulating the Aβ cleavage process.^24^^,^^52^ The results were also in line with previous studies indicating that cholesterol reduction and statin treatment promoted the α-secretase-mediated cleavage pathway of APP.^53^^,^^54^ This α-secretase-mediated pathway results in the production of α-secretase-cleaved soluble APP, a protein with neuroprotective and neurotrophic properties.^55^ Maintaining membrane fluidity could be an important factor in properly regulating the APP processing pathway.

### Effects of the composition of DHA and ARA on neuronal activity and morphology

In the presence of sufficient amounts of PUFA, the specific profiles of PUFA have been shown to impact the formation of neurites, synaptic size, synaptic density, and neuronal activity synchronization. Brain function is associated with the brain volume and morphology of each neuron.^56^^,^^57^ The present results were consistent with previous studies reporting promoted neurite outgrowth by DHA.^41^^,^^42^ While epidemiological studies have revealed that DHA intake and ARA intake are involved in maintaining brain volume,^39^^,^^40^ the present results might represent one mechanism to explain the epidemiological results, as neurite complexity is associated with brain volume size.^58^ The results of the effects of DHA and ARA on synaptic density were inconsistent with previous studies showing increased synaptic density by DHA.^41^^,^^59^ These inconsistent results might be due to differences in species and culture system, as the cultured periods of investigating neurites and synaptic sites differed between neurons differentiated from human iPSCs and primary cultured neurons from rodents. Maintaining a proper balance between synapse formation and synapse degradation for the formation of functional neuronal circuits and synaptic plasticity in the brain is crucial.^60^^,^^61^ In the present study, we observed an increase in neuronal connectivity along with decreased synaptic densities and increased synaptic sizes. It was suggested that enhanced proportions of DHA and ARA promoted synaptic modulation as the formation of neuronal networks. Since DHA and ARA have been reported to promote synaptic plasticity such as long-term potentiation,^62^^,^^63^^,^^64^ it is plausible that enhanced proportions of DHA and ARA contribute to the regulation of synapse formation in this model.

### Comparison of fatty acid composition between cultured neurons and the human brain

In the PUFA-deficient condition, MA was detected, indicating PUFA deficiency.^27^^,^^28^ On the other hand, in the PUFA-sufficient condition, MA was not present, but there were large amounts of PUFAs such as n-3 DPA, which were later replaced by the addition of DHA and ARA. We referenced and calculated the fatty acid compositions in the human brain from a previous study,^6^ and compared differences between cultured neurons and the human brain (Table S4). In both PUFA-deficient and PUFA-sufficient conditions, added DHA and ARA were incorporated into the phospholipids and decreased fatty acids that are not abundant in the human brain. It was suggested that the addition of both DHA and ARA could approximate human brain lipid characteristics in terms of fatty acid compositions. It is worth noting that PUFAs such as LA, MA, EPA, and n-3 DPA are not commonly found in the human brain, but they could be incorporated into phospholipids when levels of DHA and ARA are insufficient in cultured neurons. Although we humans are able to partially biosynthesize DHA and ARA,^7^ it seemed inadequate for cultured neurons, as shown in this study. Based on findings from the two models, it has been suggested that both the total PUFA composition and the individual types of PUFA composition are important for properly maintaining neurons. The amount of PUFA has been well studied, and it was suggested that deficiency increases the risk of diseases.^12^ The present results suggested that the profiles of PUFA are also important in neuronal function. Interestingly, this might be related to why low compositions of LA, MA, EPA, and n-3 DPA and high compositions of DHA and ARA are maintained in the human brain. Establishing a lipid composition that matches that of the human brain could result in a model that more accurately reproduces physiological conditions.

### Limitations of the study

First, we generated human neurons from human iPSCs, although we could not use human astrocytes for long-term culture in this study. The effects of lipids should be further investigated in the coculture of fully human neurons using functional human astrocytes by recently reported approaches.^65^^,^^66^ Second, it will be necessary to examine whether similar results could be obtained with neurons differentiated from other iPSC lines from healthy individuals. Moreover, iPSCs derived from patients with mutations in lipid-related genes such as ApoE, and neurological conditions affecting neuronal synchronization such as epilepsy, might help deepen our understanding of these pathological conditions. Third, we compared conditions that considerably differed from human brain lipid characteristics with conditions made closer by the additions of DHA and ARA. Lipid membrane profiles are influenced by physiological factors such as diet, aging, and neurodegenerative diseases. Further investigations will be required to determine the impact on neuronal function or Aβ cleavage processing due to various physiologically induced changes in lipid membrane composition. Fourth, our understanding of the precise molecules responsible for changes in neuronal morphology and function in DHA-/ARA-composition-enhanced conditions is still incomplete. We have not yet investigated the possibility that the free form of DHA, ARA, or their metabolites might be involved, rather than alterations in lipid membranes. There are several reports on the mechanisms of DHA action on neurite outgrowth and synapse formation. In a previous study, DHA was shown to be involved in the activation of wingless-type MMTV integration site family (WNT) signaling and cAMP Response Element Binding Protein (CREB) signaling.^42^ Another study has reported that 7-hydroxy DHA, one of the metabolites derived from DHA, promotes neurite outgrowth via PPARα.^67^ In the current study, the fatty acids in phospholipids are largely moved from n-3 DPA to DHA, which also suggests that DHA-derived metabolites might be involved because 7-hydroxy DHA could not be derived from n-3 DPA. Moreover, since all fatty acids were methylated from phospholipids and analyzed in this study, it remains unclear whether certain alterations in the phospholipid composition were influenced by specific polar head types of phospholipid, such as phosphatidylcholine, phosphatidylethanolamine, or phosphatidylinositol. Further investigations will be required to elucidate what molecules are involved in alterations of neuronal function.

To summarize, our approach, using human cortical neurons cultured under conditions of progressively alleviated PUFA deficiency, suggested that both the overall amount and the profiles of PUFAs that comprise them are essential factors that affect neuronal function and pathophysiology. This points out the importance of the unique fatty acid compositions of the human brain, where PUFAs such as DHA and ARA are abundant.

## Resource availability

### Lead contact

Further information and requests for resources and reagents should be directed to and will be fulfilled by the lead contact, Haruhisa Inoue (haruhisa@cira.kyoto-u.ac.jp).

### Materials availability

Materials used in this study were commercially available. This study did not generate new unique reagents.

### Data and code availability

• Microscopy data reported in this paper will be shared by the lead contact upon request.
• This paper does not report original code.
• Any additional information required to reanalyze the data reported in this paper is available from the lead contact upon request.

## Acknowledgments

We thank all our coworkers and collaborators for their kind support. We acknowledge Makiko Yasui, Mikie Iijima, Rumi Ono, and Akiko Kadotani for their administrative support. This study was supported by research funds under contract from Suntory Wellness Ltd., 10.13039/100009619AMED under grant numbers, JP23bm1323001 to H.I., and JP23bm1423012 and JP24wm0625501 to T.K. and H.I., and the Canon Foundation Grant Program to H.I. Portions of the figures were drawn by using pictures from Servier Medical Art; Servier is licensed under a Creative Commons Attribution 3.0 Unported License (https://creativecommons.org/licenses/by/3.0/).

## Author contributions

S.M., T.K., H.T., Y.K., and H.I. conceived the project. S.M. performed the experiments. S.M., T.K., H.T., Y.K., T.I., Y.N., and H.I. analyzed the data and provided scientific discussions. S.M., T.K., and H.I. wrote the manuscript.

## Declaration of interests

S.M., H.T., Y.K., T.I., and Y.N. are employees of Suntory Wellness Ltd., which markets health food products that include PUFAs.

## STAR★Methods

### Key resources table

**Table:** 

REAGENT or RESOURCE	SOURCE	IDENTIFIER
**Antibodies**		

Mouse monoclonal anti-Beta-III Tubulin	Merck	Cat# MAB1637; RRID:AB_2210524
Chicken polyclonal anti-MAP2	Abcam	Cat# ab5392; RRID:AB_2138153
Rabbit polyclonal anti-GFAP	Agilent	Cat# Z0334; RRID:AB_10013382
Rabbit polyclonal anti-Synapsin 1	Merck	Cat# AB1543; RRID:AB_2200400
Mouse monoclonal anti-PSD-95	Abcam	Cat# ab2723; RRID:AB_303248
Rabbit polyclonal anti-EEA1	Thermo Fisher Scientific	Cat# PA1-063A; RRID:AB_2096819
Goat anti-Mouse, Alexa Fluor 488	Thermo Fisher Scientific	Cat# A-32723; RRID:AB_2633275
Goat anti-Rabbit, Alexa Fluor 546	Thermo Fisher Scientific	Cat# A-11010; RRID:AB_2534077
Goat anti-Chicken, Alexa Fluor 647	Thermo Fisher Scientific	Cat# A-21449; RRID:AB_2535866

**Chemicals, peptides, and recombinant proteins**		

StemFit AK02N	Ajinomoto	Cat# RCAK02N
iMatrix-511	nippi	Cat# 892012
DMEM/F-12, GlutaMAX™ supplement	Thermo Fisher Scientific	Cat# 10565018
Neurobasal™ Medium	Thermo Fisher Scientific	Cat# 21103049
Neurobasal™ Plus Medium	Thermo Fisher Scientific	Cat# A3582901
AGM™ Astrocyte Growth Medium BulletKit™	Lonza	Cat# CC-3186
B-27™ Plus Supplement	Thermo Fisher Scientific	Cat# A3582801
B-27™ Supplement, minus vitamin A	Thermo Fisher Scientific	Cat# 12587010
KnockOut Serum Replacement (KSR)	Thermo Fisher Scientific	Cat# 10828028
GlutaMAX	Thermo Fisher Scientific	Cat# 35050-061
Matrigel	Corning	Cat# 354234
Synthemax II-SC	Corning	Cat# 3535
CellNest	FujiFilm	Cat# 16461438
Poly-l-lysine	Merck	Cat# P4707
Doxycyclin	Takara	Cat# 631311
G418 Disulfate	Nacalai Tesque	Cat# 09380-86
MEM Non-Essential Amino Acids Solution	Thermo Fisher Scientific	Cat# 11140050
TrypLE™ Select Enzyme	Thermo Fisher Scientific	Cat# 12563011
2-Mercaptoethanol	Thermo Fisher Scientific	Cat# 21985023
BDNF	Thermo Fisher Scientific	Cat# 450-02
GDNF	Thermo Fisher Scientific	Cat# 450-10
NT-3	Thermo Fisher Scientific	Cat# 450-03
SB431542	Cayman Chemical	Cat# 13031
Dorsomorphin	Merck	Cat# P5499
Y-27632	Nacalai Tesque	Cat# 18188-04
cOmplete™ mini, EDTA-free Protease inhibitor cocktail tablets	Roche	Cat# 11836170001
cis-4,7,10,13,16,19-docosahexaenoic acid	Merck	Cat# D2534
Arachidonic acid	Merck	Cat# A3611
Normal Goat Serum	Abcam	Cat# ab7481
DAPI	Thermo Fisher Scientific	Cat# 62248
Methyl-β-cyclodextrin	Merck	Cat# 332615
β-secretase inhibitor	Merck	Cat# 565788
γ-secretase inhibitor	Merck	Cat# SML0683
LipiORDER	Funakoshi	Cat# FDV-0041

**Critical commercial assays**		

V-PLEX Plus Aβ Peptide Panel 1 (6E10) Kit	MSD	Cat# K15200G-2
Cholesterol/Cholesterol Ester-Glo™ Assay	Promega	Cat# J3190
Pierce BCA Protein Assay Kit	Thermo Fisher Scientific	Cat# 23227

**Experimental models: Cell lines**		

Brain mixed astrocytes	Lonza	Cat# M-ASM-330
Human iPSC	RIKEN BioResource Research Center	HPS1043
Human iPSC	RIKEN BioResource Research Center	HPS1046

**Software and algorithms**		

ImageJ	NIH	Version 1.52a
SPSS	IBM	Version 26
Prism Software	Graphpad	Version 10.4

### Experimental model and study participant details

#### Human iPSC maintenance

The use of iPSC was approved by the Ethics Committees of RIKEN BioResource Research Center. Human iPSC was provided by the RIKEN BioResource Research Center through the National BioResource Project of the MEXT, Japan. Human iN-iPSC, containing doxycycline-inducible human NGN2 construct, was used as previously described.^38^ The data on human iPSCs used in the present study is shown in Key resources table. iPSC was maintained on laminin (iMatrix-511; TAKARA, Kusatsu, Japan) in StemFit AK02N (Ajinomoto, Tokyo, Japan) at 37°C in a 5% CO_2_ incubator, as previously described,^68^ with slight modifications. iN-iPSC was maintained in StemFit AK02N containing 50 μg/mL G418 disulfate (Nacalai Tesque, Kyoto, Japan). Passages were performed every seventh day. Cells were dissociated using TrypLE select enzyme (Thermo Fisher Scientific Inc., Waltham, MA, USA) and cultured in StemFit AK02N with 10 μM Y-27632 (Nacalai Tesque). The medium was changed to remove Y-27632 on the following day.

### Method details

#### Differentiation of neurons from human iPSC

Neurons were differentiated from iPSCs as previously described.^69^ iPSC was dissociated with TrypLE select enzyme and placed in U-bottom 96 well plate (Thermo Fisher Scientific) to form embryonic bodies (EB) for suspension culture. From day 0 to day 8, EBs were cultured in DMEM/F12 with GlutaMAX (Thermo Fisher Scientific) containing 5% KSR (Thermo Fisher Scientific), NEAA (Thermo Fisher Scientific), 0.1 M 2-mercaptoethanol (Thermo Fisher Scientific) with 10 μM Y-27632, 2 μM dorsomorphin (Thermo Fisher Scientific) and 10 μM SB431542 (Thermo Fisher Scientific). After neuronal induction, EBs were transferred to matrigel (Corning, NY, USA) -coated plates and cultured in DMEM/F12 with GlutaMAX containing 1% N-2 supplement (Thermo Fisher Scientific), 5% KSR, NEAA, 0.1 M 2-mercaptoethanol (Thermo Fisher Scientific) with 2 μM dorsomorphin from day 8 to day 22. On day 22, mitigated neuronal precursor cells were dissociated with TrypLE select enzyme and cultured in Neurobasal Medium (Thermo Fisher Scientific) containing B-27 without Vitamin A (Thermo Fisher Scientific), GlutaMAX, 10 ng/mL BDNF (Thermo Fisher Scientific), 10 ng/mL GDNF (Thermo Fisher Scientific), 10 ng/mL NT-3 (Thermo Fisher Scientific) on matrigel-coated plates for neuronal maturation. Half the culture medium was changed twice a week.

#### Neuron – Astrocyte coculture

Brain mixed astrocytes were purchased from LONZA (M-ASM-330; Basel, Switzerland) and cultured according to the manufacturer’s instructions. Human neurons used for neuron–astrocyte coculture were differentiated using direct conversion technology as previously described.^38^ On day 0, iN-iPSC was dissociated with TrypLE select enzyme and placed on a mixed coating of poly-L-lysine (0.0002% w/v; Merck, Darmstadt, Germany), Matrigel (2% v/v; Corning), Synthemax 2-SC (20 μg/mL; Corning), CellNest (2% v/v; FujiFilm, Tokyo, Japan) in Neurobasal plus medium containing 2% B-27 plus supplement (Thermo Fisher Scientific), 2 μg/mL doxycycline (FujiFilm), 10 μM Y-27632, and antibiotics. On day 5, differentiated neurons were dissociated and placed on cultured brain mixed astrocytes and maintained in Neurobasal plus medium containing 2% B-27 plus supplement, 10 μM Y-27632, and antibiotics. On day 8, the medium was replaced with Neurobasal plus medium containing 2% B-27 plus supplement and each of the fatty acids.

#### Compound treatment in cultured cells

Docosahexaenoic acid (Merck), and arachidonic acid (Merck) were dissolved in dimethyl sulfoxide (Nacalai Tesque) and added to the medium at a final concentration of 0.1% v/v. Half the culture medium was changed twice a week.

#### Immunocytochemistry (ICC)

Cells were fixed in 4% paraformaldehyde (PFA) (Nacalai Tesque) for 10 min at room temperature, permeabilized in 0.1 % Triton X-100 (Nacalai Tesque) for 10 min at room temperature, and incubated with 5% Goat Serum (Abcam, Cambridge, UK). Incubation with primary antibodies was performed at 4°C overnight. After incubation with secondary antibodies for 1 hour at room temperature, DAPI (Thermo Fisher Scientific) was incubated for 10 min at room temperature. Cell Images were acquired using Operaphenix (Perkin Elmer, Waltham, MA, USA) and analyzed with Harmony software (Perkin Elmer). The primary and secondary antibodies used in the assay were listed in the Key resources table. To quantify the proportions of MAP2-positive and GFAP-positive cells, we counted the total number of nuclei (DAPI-positive), nuclei within MAP2-immunoreactive regions, and nuclei within GFAP-immunoreactive regions. For the analysis of synaptic sites, we calculated the area (μm^2^) of neuronal regions within the imaging field using MAP2 signals. Next, we detected synapsin and PSD-95 signals present within the identified neuronal regions and calculated their respective spot counts and spot sizes. Furthermore, we identified overlapping spots between the detected synapsin and PSD-95 signals, and similarly calculated their spot counts and spot sizes. The spot counts for synapsin, PSD-95, and merged signals were normalized by the neuronal region area (μm^2^) within the imaging field and calculated as density. We performed similar analyses across different acquired images. For neurite analysis, the process consisted of three primary steps: first, neuronal regions within the imaging field were identified through MAP2 signal detection; second, nuclear identification was performed using DAPI signals, with neuronal nuclei being differentiated from astrocytic nuclei based on their characteristically larger size; third, neurite detection and parameter quantification were conducted based on the identified neuronal nuclei and their corresponding regions.

#### MEA recordings of cultured cells

MEAs were recorded and analyzed as described previously.^34^^,^^65^^,^^70^ Cells were cultured using the Cytoview MEA plates with 16 electrodes per well (Axion Biosystems, Atlanta, GA, USA). Cytoview MEA plates were coated with poly-L-lysine (0.0002% w/v), Matrigel (2% v/v), Synthemax 2-SC (20 μg/mL), and CellNest (2% v/v). Spontaneous neuronal activities were recorded with Maestro System (Axion Biosystems) sampling at 12.5 kHz, filtering, and spike detection, delivering whole channel neuronal spike data. Recordings were made after being treated with fatty acids for 4, 8, 12, and 16 weeks. MEA cultures were maintained at 37°C, activities were measured for 10 min, and data from the last 5 min was used for analysis. Analysis of recorded data was performed using Neuronal Metric Tool (Axion Biosystems). Active electrodes were defined as electrodes averaging more than 5 spikes per minute. Active wells were defined as those with more than 5 active electrodes. All wells not complying with these criteria were discarded. For calculation of the synchronicity of the neuronal culture, bursts with at least 25% electrode participation in a well were defined as network bursts.

#### Fatty acid analysis

Cells were dissociated using TrypLE select enzyme, suspended in PBS, and centrifuged at 400 g for 5 min. Methanol (Nacalai Tesque) was added to the collected pellets and stored at -80°C prior to fatty acid analysis. Lipids in cells were extracted and purified by the method of Bligh and Dyer.^71^ The phospholipid fraction was separated by thin-layer chromatography (Merck) with hexane:ether = 7:3 (Nacalai Tesque) and incubated with methanolic hydrochloric acid (Nacalai Tesque) at 50°C for 3 h for transmethylation of fatty acid residues. Fatty acid methyl esters were extracted with n-hexane (Nacalai Tesque) and analyzed by gas chromatography (Agilent 7890B, Agilent Technologies, Santa Clara, CA, USA) as described previously.^9^ The composition of each fatty acid was expressed as a percentage of the total peak area of the identified fatty acids. We obtained the relative levels of individual fatty acids, expressed as the percent of total fatty acids in phospholipids, including: SFAs (16:0, 18:0, 20:0, 22:0, 24:0), MUFAs (16:1, 18:1 n-9, 18:1 n-7, 20:1, 22:1, 24:1), PUFAs (18:2 n-6, 18:3 n-6, 18:3 n-3, 20:2 n-6, 20:3 n-9, 20:3 n-6, 20:4 n-6, 20:5 n-3, 22:4 n-6, 22:5 n-3, 22:6 n-3). Fatty acid concentrations in culture medium were analyzed by the same method.^71^ Pentadecanoic acid (15:0) was used as internal control and extracted total fatty acids were analyzed by gas chromatography. Each fatty acid concentration in the culture medium was calculated by its ratio with the internal control. Brain fatty acid concentrations were estimated using an age-specific formula, with calculations based on an assumed age of 60 years.^6^

#### Membrane fluidity measurement

Membrane fluidity of neurons was measured using LipiORDER (Funakoshi, Tokyo, Japan) according to the manufacturer’s instructions. Briefly, cells were incubated with 1 μM LipiORDER for 30 min at 37°C, 5% CO_2_. After removing LipiORDER, live imaging was conducted using CellVoyager CV8000 (Yokogawa Electric, Tokyo, Japan) at 37°C, 5% CO_2_. LipiORDER was exited at 405 nm, and its fluorescence was obtained using 525 nm channel and 600 nm channel in sequence. The ratios of 600 nm intensity and 525 nm intensity were calculated by ImageJ (NIH, Bethesda, MD, USA) as previously described.^72^ For the analysis of neuronal or endosomal membrane fluidity, we performed live imaging using LipiORDER and fixed cells immediately by paraformaldehyde. After immunostaining for neuronal or endosome markers, we re-imaged the same fields of view. We overlaid the ratio image of Intensity 600/Intensity 515 with the neuronal regions to calculate neuron-specific fluidity.

#### Total protein concentration measurement

Cells were lysed in RIPA buffer (FujiFilm, Tokyo, Japan) with cOmplete™ Mini (Roche) and the supernatant was collected after centrifugation (4°C, 10,000 g, 10 min). Total protein concentration was determined using the BCA protein assay kit (Thermo Fisher Scientific) and absorption at 562 nm was analyzed by ENVISION (Perkin Elmer).

#### Cholesterol measurement

Cholesterol concentrations were analyzed using a Cholesterol GMO assay kit (Promega, Madison, WI, USA) following the manufacturer’s instructions. Luminescence was analyzed by ENVISION (Perkin Elmer). Relative luminescence levels were normalized by the total protein concentrations.

#### Aβ measurement

The culture supernatant was collected after centrifugation (400 g, 10 min) and stored at -80°C prior to analysis. Aβ in the culture supernatant was measured using Human (6E10) Aβ 3-Plex Kit (Meso Scale Discovery, Rockville, MD, USA) according to the manufacturer’s instructions. Electrochemiluminescence was detected by Sector Imager 6000 (Meso Scale Discovery). The Aβ concentrations were normalized by the total protein concentrations.

### Quantification and statistical analysis

#### Statistical analysis

Data are expressed as mean ± standard error (SE). Results were analyzed using Mann-Whitney test or Kruskal-Wallis test followed by Dunn’s multiple comparisons test for nonparametric analysis, and unpaired t-test or Dunnett’s test for parametric analysis to determine the statistical significance of the data compared to control. All analysis was performed using SPSS statistics 26 (SPSS, Inc., Chicago, IL, USA) and Prism 9 (GraphPad Software, Boston, MA, USA). All statistical test results are shown in Table S5.

## Supplemental information

Document S1. Figures S1–S7 and Tables S1–S4

Table S5. Excel file containing additional data too large to fit in a PDF, related to STAR Methods
