# Supplementary material for: Polyunsaturated fatty acids in lipid membranes regulate human neuronal function and amyloid-β production
Source: iScience. 2025 May 12;28(6):112557. doi: 10.1016/j.isci.2025.112557 (PMC12152501; doi:10.1016/j.isci.2025.112557)
Supplement: Document S1. Figures S1–S7 and Tables S1–S4 [file mmc1.pdf]

iScience, Volume *28*

Supplemental information

Polyunsaturated fatty acids in lipid membranes regulate human neuronal function and amyloid-β production

Satoshi Morita, Takayuki Kondo, Hisanori Tokuda, Yoshihisa Kaneda, Takayuki Izumo, Yoshihiro Nakao, and Haruhisa Inoue

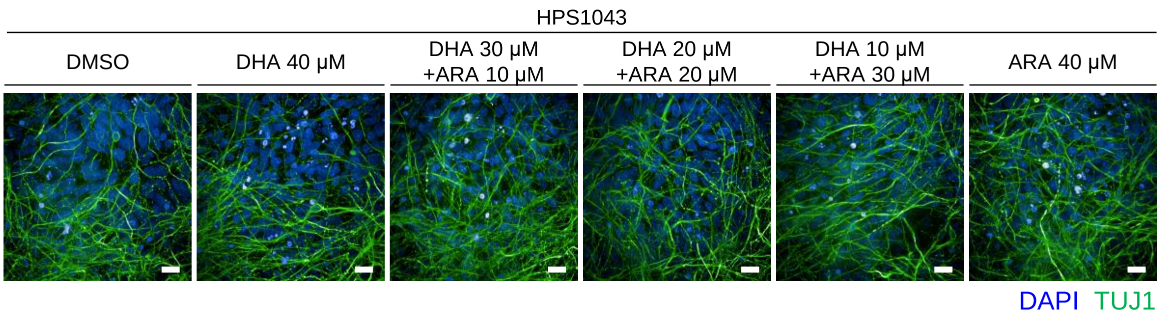
HPS1043
DMSO
DHA 40 μM
DHA 30 μM +ARA 10 μM
DHA 20 μM +ARA 20 μM
DHA 10 μM +ARA 30 μM
ARA 40 μM
DAPI
TUJ1

A

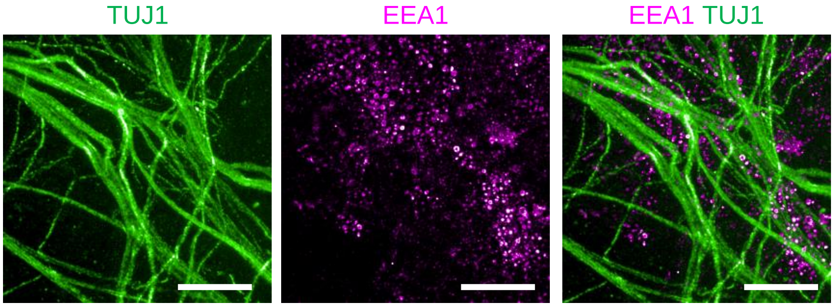
TUJ1
EEA1
EEA1 TUJ1

B

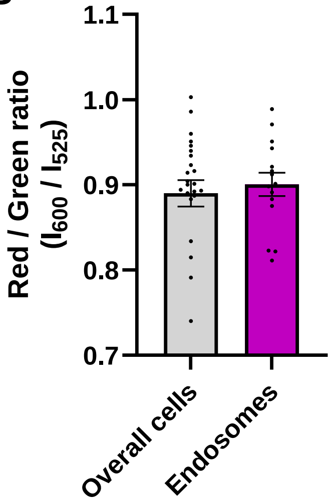
1.1
1.0
0.9
0.8
0.7
Red / Green ratio
(I600 / I525)
Overall cells
Endosomes

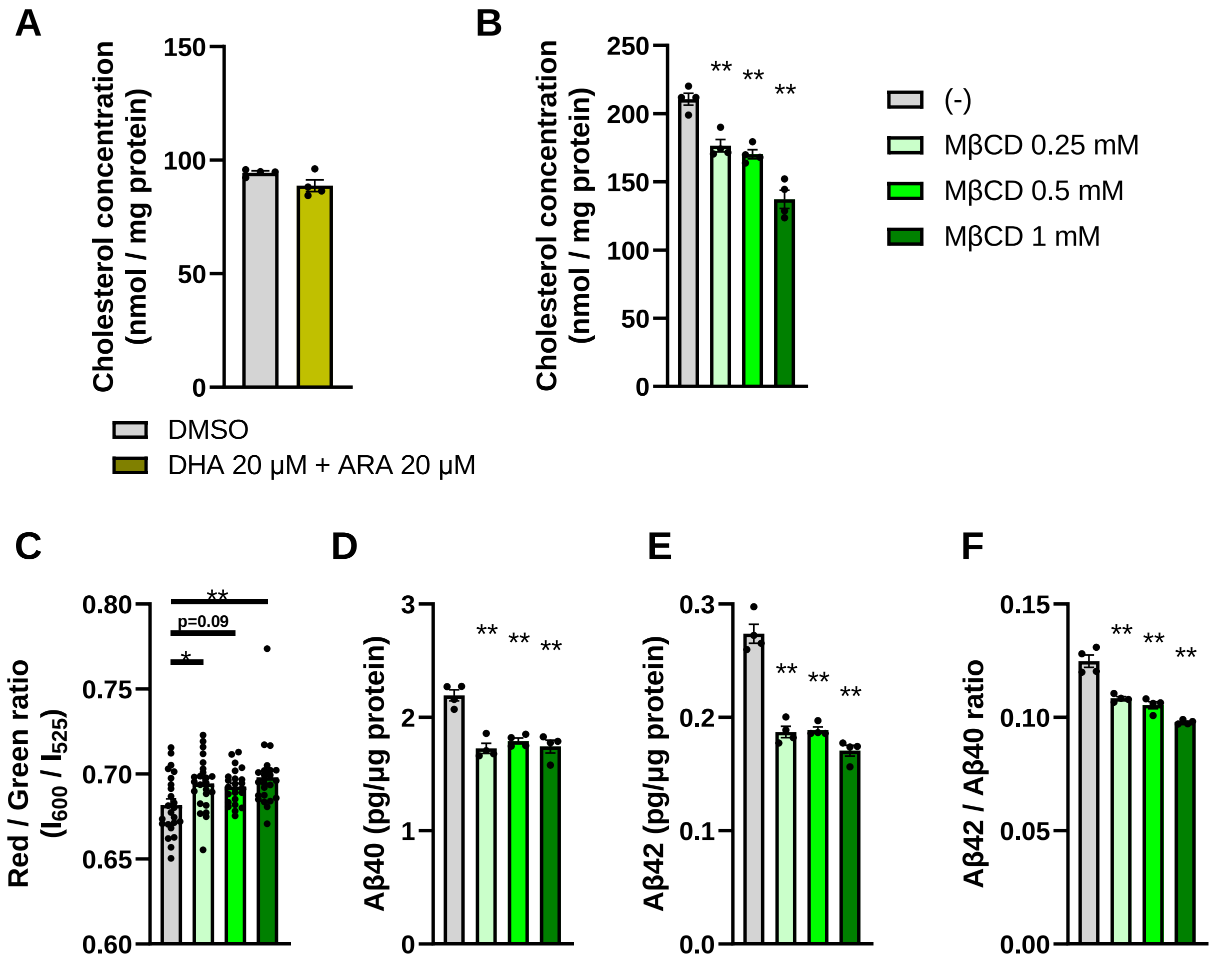

A
Cholesterol concentration (nmol / mg protein)
150
100
50
0
DMSO
DHA 20 μM + ARA 20 μM
B
Cholesterol concentration (nmol / mg protein)
250
200
150
100
50
0
** ** **
(-)
MβCD 0.25 mM
MβCD 0.5 mM
MβCD 1 mM
C
Red / Green ratio (I600 / I525)
0.80
0.75
0.70
0.65
0.60
**
p=0.09
*
D
Aβ40 (pg/μg protein)
3
2
1
0
** ** **
E
Aβ42 (pg/μg protein)
0.3
0.2
0.1
0.0
** ** **
F
Aβ42 / Aβ40 ratio
0.15
0.10
0.05
0.00
** ** **

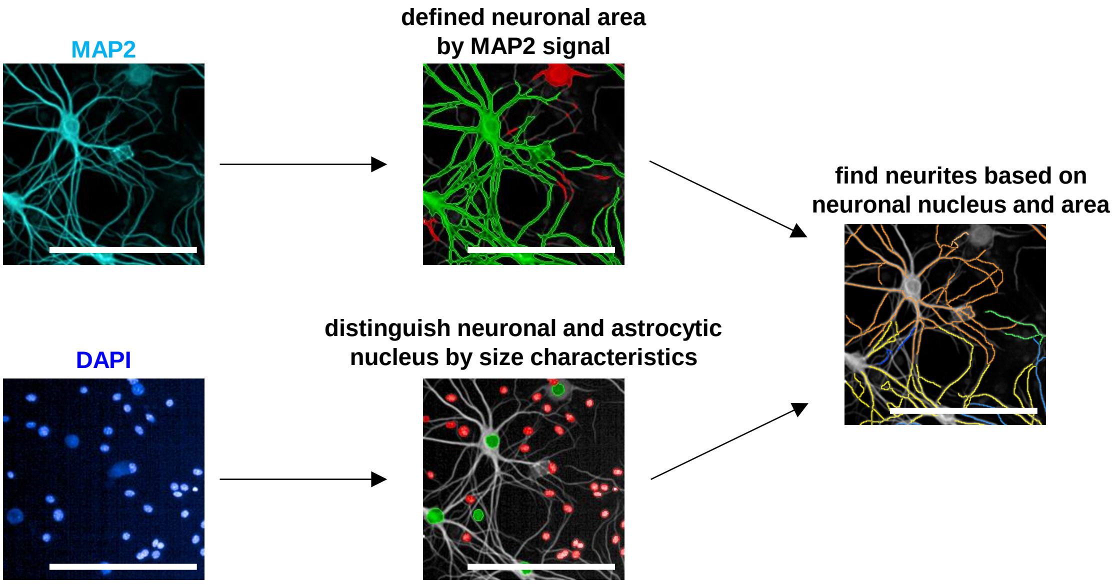
MAP2
defined neuronal area
by MAP2 signal
find neurites based on
neuronal nucleus and area
DAPI
distinguish neuronal and astrocytic
nucleus by size characteristics

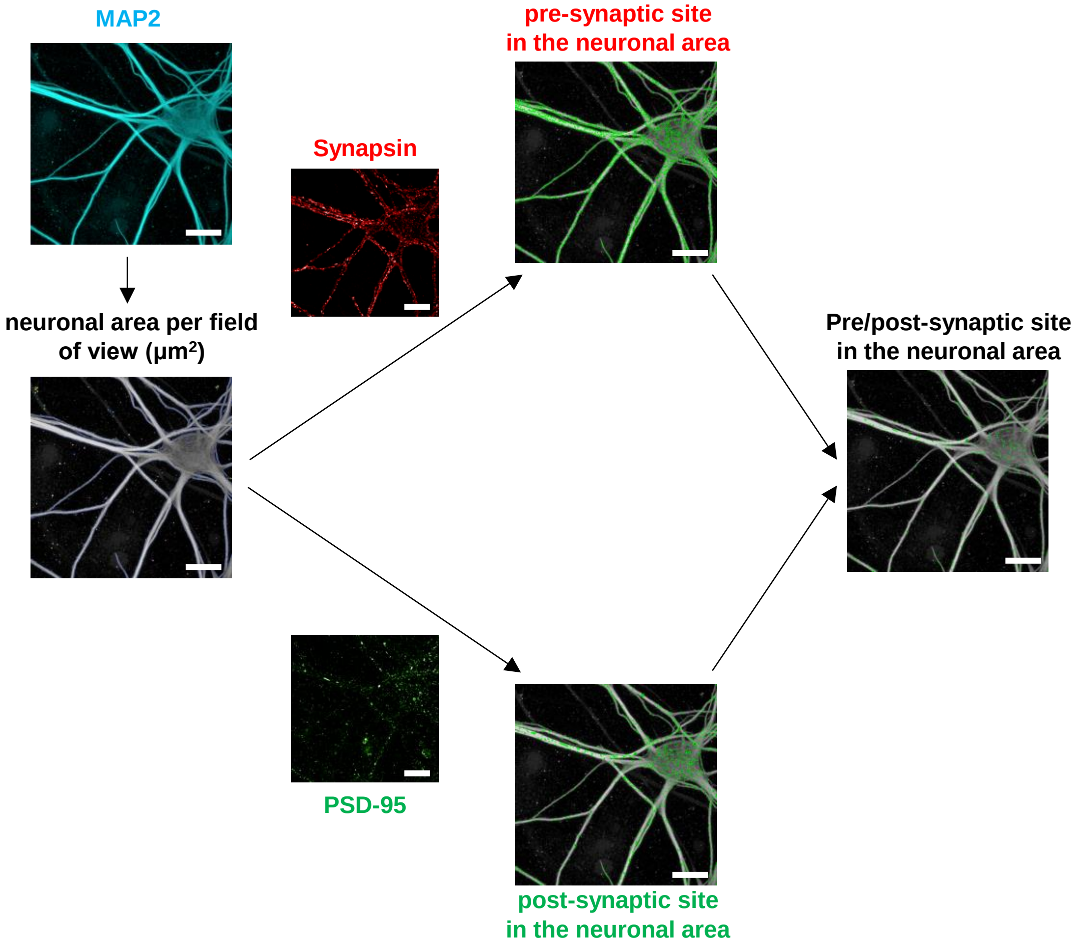
MAP2
neuronal area per field of view (μm²)
Synapsin
PSD-95
pre-synaptic site in the neuronal area
post-synaptic site in the neuronal area
Pre/post-synaptic site in the neuronal area

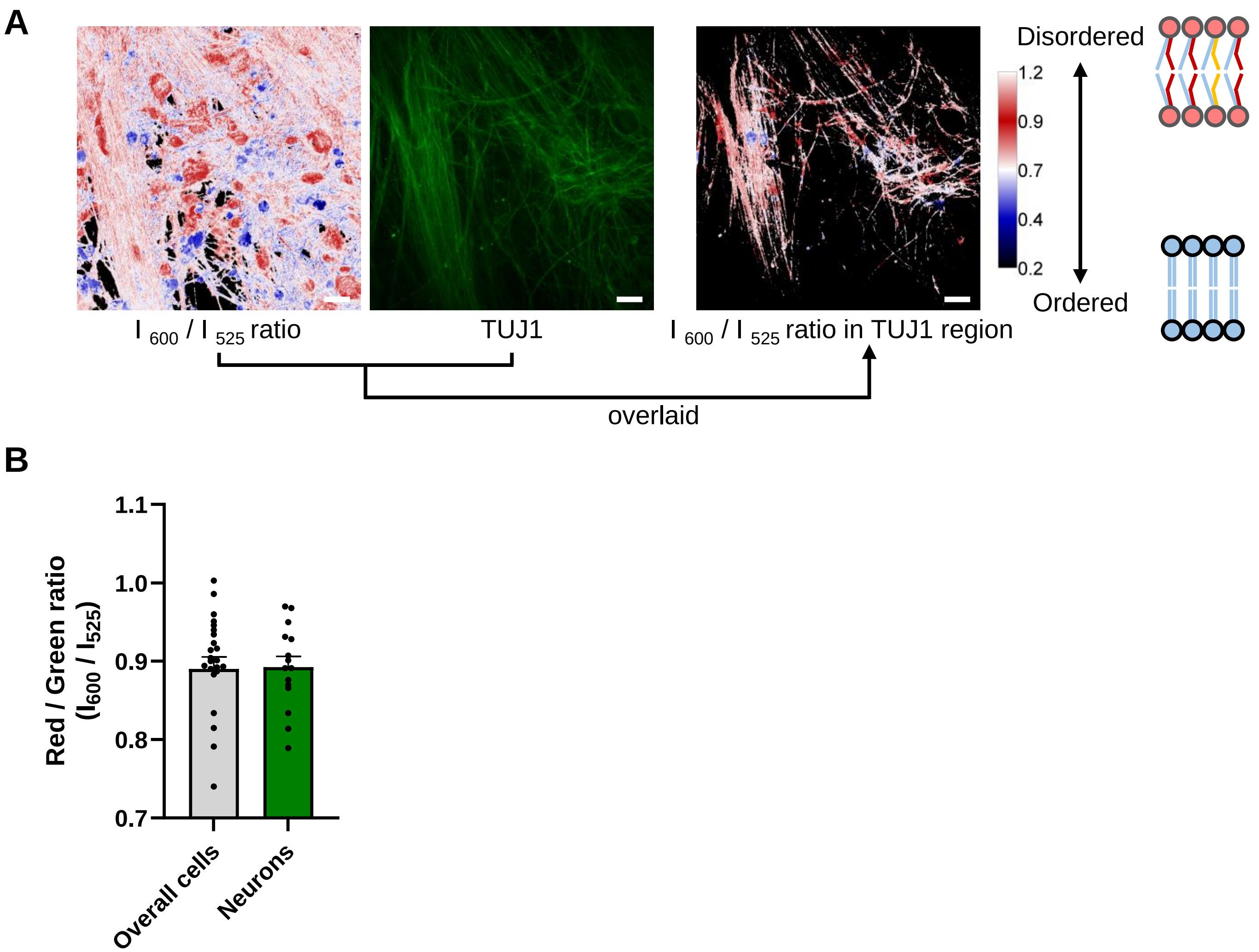

A
Disordered
1.2
0.9
0.7
0.4
0.2
Ordered
I 600 / I 525 ratio
TUJ1
I 600 / I 525 ratio in TUJ1 region
overlaid
B
1.1
1.0
0.9
0.8
0.7
Red / Green ratio
(I600 / I525)
Overall cells
Neurons

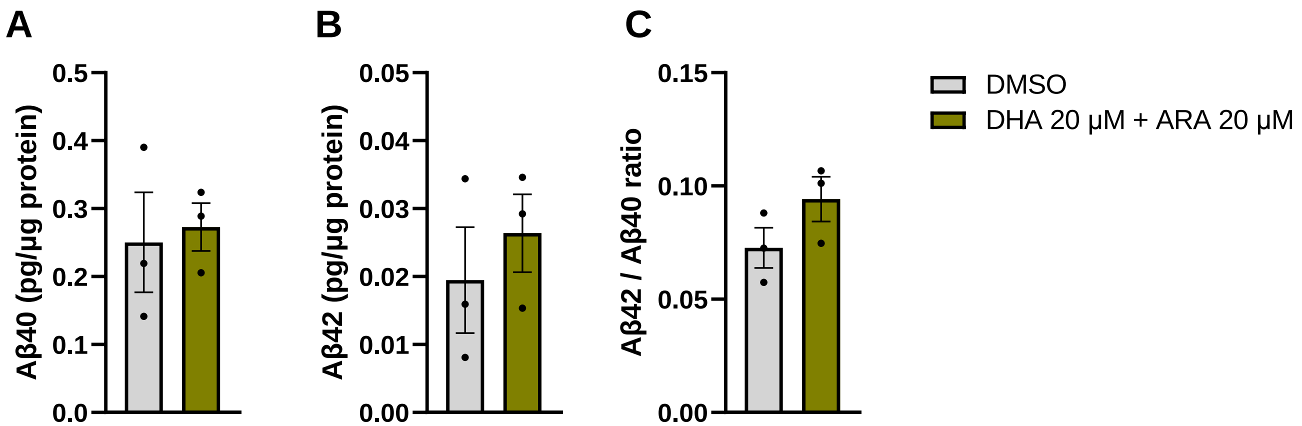
A
B
C
Aβ40 (pg/µg protein)
0.0
0.1
0.2
0.3
0.4
0.5
Aβ42 (pg/µg protein)
0.00
0.01
0.02
0.03
0.04
0.05
Aβ42 / Aβ40 ratio
0.00
0.05
0.10
0.15
DMSO
DHA 20 µM + ARA 20 µM

Supplemental figure legends

Figure S1. iPSC-derived neurons after addition of DHA and/or ARA for 4 weeks.

Representative images of iPSC-derived neurons after addition of DHA and/or ARA for 4 weeks. Scale bars, 20 µm.

Figure S2. Endosomal lipid membrane fluidity of cells treated with DHA and ARA for 4 weeks.

(A) The Red / Green ratio was overlaid with the EEA1 region. Scale bars, 25 µm.

(B) The Red / Green ratio in the EEA1 region was calculated for each picture. Data represent mean ± SE, 15 or 24 images were analyzed from 3 independent biological samples for each condition.

Figure S3. Cholesterol depletion increased lipid membrane fluidity and reduced Aβ40 and Aβ42 production.

(A) The amount of cellular free cholesterol was analyzed after being treated with DHA and ARA for 4 weeks. Data represent mean ± SE, n = 4.

(B) The amount of cellular free cholesterol was analyzed after being treated with methyl-β-cyclodextrin (MβCD) for 48 h. Data represent mean ± SE, n = 4. Dunnett's test compared to control, $*p < 0.05$, $**p < 0.01$.

(C) The Red / Green ratio was calculated for each picture. Data represent mean ± SE, 24 images were analyzed from 4 independent biological samples for each condition. Kruskal-Wallis test followed by Dunn's test compared to DMSO control, $*p < 0.05$, $**p < 0.01$.

(D-F) The amount of Aβ40 (D), Aβ42 (E), and the ratio of Aβ42 / Aβ40 (F) in a 48-h cultured medium was analyzed. Mean ± SE, n = 4. Dunnett's test compared to control, $*p < 0.05$, $**p < 0.01$.

Figure S4. Analytical workflow for neurite characterization in neuron-astrocyte coculture system

Neurite morphology was analyzed following the workflow. Scale bars, 200 µm.

Figure S5. Analytical workflow for synaptic characterization in neuron-astrocyte coculture system

Synaptic density and size parameters were analyzed following the workflow. Scale bars, 20 µm.

Figure S6. Neuronal lipid membrane fluidity of cells treated with DHA and ARA for 4 weeks.

(A) The Red / Green ratio was overlaid with neuronal region. Scale bars, 20 µm.

(B) The Red / Green ratio in neuronal region was calculated for each picture. Data represent mean ± SE, 15 or

24 images were analyzed from 3 independent biological samples for each condition.

Figure S7. Aβ40 and Aβ42 productions were not altered under stable membrane fluidity.

(A-C) The amount of Aβ40 (A), Aβ42 (B), and the ratio of Aβ42 / Aβ40 (C) in a 48-h cultured medium was analyzed. DHA and ARA were treated for 4 weeks. Data represent mean ± SE, n = 3.

Table S1. Fatty acid concentrations (μM) in culture medium medium with LA and ALA as exclusive PUFA sources, related to Figure 1. n.d. = not detected.

Fatty acids (Number of carbons : double bonds)		Mean ± SE (μM)
Palmitic acid	(16:0)	0.03 ± 0.00
Stearic acid	(18:0)	0.10 ± 0.00
Oleic acid	(18:1)	0.02 ± 0.00
Linoleic acid (LA)	(18:2)	3.29 ± 0.07
α-Linolenic acid	(18:3)	0.85 ± 0.02
Mead acid	(20:3)	n.d.
Dihomo-γ-Linoenic acid (DGLA)	(20:3)	n.d.
Arachidonic acid (ARA)	(20:4)	n.d.
Eicosapentaenoic acid (EPA)	(20:5)	n.d.
Adrenic acid (AdrA)	(22:4)	n.d.
n-3 Docosapentaenoic acid (n-3 DPA)	(22:5)	n.d.
Docosahexaenoic acid (DHA)	(22:6)	n.d.
Others		n.d.
Saturated fatty acids (SFA)		0.13 ± 0.01
Monounsaturated fatty acids (MUFA)		0.02 ± 0.00
Polyunsaturated fatty acids (PUFA)		4.15 ± 0.08

Table S2. Fatty acid compositions in phospholipids (%) in cells cultured for 4 weeks with DHA and/or ARA, related to Figure 1. n.d. = not detected.

Fatty acids<br>(Number of carbons : double bonds)		DMSO	DHA 40 μM	DHA 30 μM +<br>ARA 10 μM	DHA 20 μM +<br>ARA 20 μM	DHA 10 μM +<br>ARA 30 μM	ARA 40 μM
Palmitic acid	(16:0)	27.4 ± 0.3	27.6 ± 0.1	27.8 ± 0.1	29.4 ± 0.7	29.5 ± 0.1	33.0 ± 0.3
Palmitoleic acid	(16:1)	3.3 ± 0.0	1.9 ± 0.1	1.7 ± 0.0	1.9 ± 0.4	1.4 ± 0.0	1.0 ± 0.0
Stearic acid	(18:0)	12.9 ± 0.8	19.2 ± 0.1	18.3 ± 0.1	17.6 ± 0.2	16.3 ± 0.2	14.4 ± 1.7
Oleic acid	(18:1)	23.8 ± 0.4	9.5 ± 0.3	7.9 ± 0.1	7.5 ± 1.3	6.2 ± 0.3	3.9 ± 0.2
Vaccenic acid	(18:1)	8.2 ± 0.1	2.2 ± 0.0	2.1 ± 0.0	1.9 ± 0.1	1.9 ± 0.0	1.5 ± 0.0
Linoleic acid	(18:2)	2.5 ± 0.1	4.8 ± 0.2	4.5 ± 0.1	4.3 ± 0.2	3.9 ± 0.1	3.4 ± 0.1
α-Linolenic acid	(18:3)	0.1 ± 0.0	0.5 ± 0.0	0.4 ± 0.0	0.4 ± 0.0	0.3 ± 0.0	0.3 ± 0.0
Mead acid	(20:3)	1.6 ± 0.0	n.d.	n.d.	n.d.	n.d.	n.d.
Dihomo-γ-Linoenic acid	(20:3)	3.4 ± 0.2	1.4 ± 0.0	2.5 ± 0.0	3.4 ± 0.3	4.9 ± 0.1	7.8 ± 0.4
Arachidonic acid	(20:4)	6.0 ± 0.1	0.9 ± 0.0	10.7 ± 0.1	13.2 ± 1.0	16.2 ± 0.2	18.4 ± 0.6
Eicosapentaenoic acid	(20:5)	0.7 ± 0.3	8.3 ± 0.1	3.6 ± 0.0	1.9 ± 0.1	1.0 ± 0.0	n.d.
Adrenic acid	(22:4)	2.9 ± 0.1	0.1 ± 0.0	1.6 ± 0.0	2.9 ± 0.3	5.0 ± 0.1	9.4 ± 0.5
n-3 Docosapentaenoic acid	(22:5)	2.5 ± 0.1	3.2 ± 0.0	2.4 ± 0.0	1.8 ± 0.2	1.4 ± 0.0	0.6 ± 0.0
Docosahexaenoic acid	(22:6)	1.8 ± 0.1	19.2 ± 0.3	14.8 ± 0.1	11.0 ± 0.8	8.3 ± 0.1	0.5 ± 0.0
Others		2.9 ± 0.1	1.1 ± 0.1	1.7 ± 0.0	2.7 ± 0.2	3.6 ± 0.1	5.9 ± 0.3

Table S3. Fatty acid concentrations (μM) in culture medium for PUFA-sufficient condition, related to Figure 3. n.d. = not detected.

Fatty acids (Number of carbons : double bonds)		Mean ± SE (μM)
Palmitic acid	(16:0)	19.04 ± 0.17
Stearic acid	(18:0)	18.17 ± 0.18
Oleic acid	(18:1)	24.37 ± 0.27
Linoleic acid (LA)	(18:2)	10.53 ± 0.20
α-Linolenic acid	(18:3)	6.45 ± 0.06
Mead acid	(20:3)	n.d.
Dihomo-γ-Linoenic acid (DGLA)	(20:3)	0.72 ± 0.01
Arachidonic acid (ARA)	(20:4)	0.91 ± 0.01
Eicosapentaenoic acid (EPA)	(20:5)	0.85 ± 0.01
Adrenic acid (AdrA)	(22:4)	0.06 ± 0.00
n-3 Docosapentaenoic acid (n-3 DPA)	(22:5)	0.91 ± 0.01
Docosahexaenoic acid (DHA)	(22:6)	0.15 ± 0.00
Others		2.71 ± 0.03
Saturated fatty acids (SFA)		37.29 ± 0.35
Monounsaturated fatty acids (MUFA)		26.65 ± 0.29
Polyunsaturated fatty acids (PUFA)		20.86 ± 0.30

Table S4. Fatty acid compositions in phospholipids (%) in cells cultured for 16 weeks with DHA and/or ARA, related to Figure 7. Fatty acid compositions of the human frontal cerebral cortex were referenced from the previous study [6]. n.d. = not detected.

Fatty acids (Number of carbons : double bonds)		DMSO	DHA 20 μM	ARA 20 μM	DHA 10 μM + ARA 10 μM	DHA 20 μM + ARA 20 μM	Human brain [6]
Palmitic acid	(16:0)	18.1 ± 1.0	19.7 ± 0.7	18.1 ± 2.2	18.4 ± 0.4	19.7 ± 0.4	22.4
Palmitoleic acid	(16:1)	0.7 ± 0.0	0.6 ± 0.1	0.5 ± 0.0	0.8 ± 0.1	0.6 ± 0.0	
Stearic acid	(18:0)	26.6 ± 1.1	25.4 ± 1.5	28.5 ± 1.6	24.4 ± 0.9	21.1 ± 0.2	23.9
Oleic acid	(18:1)	14.7 ± 0.5	14.1 ± 0.7	12.0 ± 1.0	14.3 ± 1.4	14.1 ± 0.1	18.6
Vaccenic acid	(18:1)	1.9 ± 0.1	1.6 ± 0.1	1.6 ± 0.1	1.7 ± 0.0	1.7 ± 0.0	
Linoleic acid	(18:2)	3.6 ± 0.3	3.1 ± 0.2	1.7 ± 0.2	2.0 ± 0.1	1.7 ± 0.1	1.0
α-Linolenic acid	(18:3)	0.3 ± 0.0	0.1 ± 0.1	0.3 ± 0.0	0.2 ± 0.1	0.1 ± 0.1	
Mead acid	(20:3)	n.d.	n.d.	n.d.	n.d.	n.d.	0.1
Dihomo-γ-Linoenic acid	(20:3)	0.9 ± 0.1	1.1 ± 0.2	0.3 ± 0.1	0.4 ± 0.1	0.4 ± 0.0	1.0
Arachidonic acid	(20:4)	12.0 ± 0.7	5.5 ± 0.4	19.9 ± 1.5	14.2 ± 1.1	15.1 ± 0.5	9.4
Eicosapentaenoic acid	(20:5)	1.7 ± 0.1	1.6 ± 0.2	n.d.	0.2 ± 0.1	0.1 ± 0.1	
Adrenic acid	(22:4)	2.1 ± 0.1	0.2 ± 0.2	6.5 ± 0.3	3.0 ± 0.4	4.3 ± 0.2	4.9
n-3 Docosapentaenoic acid	(22:5)	13.6 ± 0.5	2.0 ± 0.3	8.0 ± 0.6	1.9 ± 0.1	1.5 ± 0.0	0.3
Docosahexaenoic acid	(22:6)	3.3 ± 0.1	24.3 ± 0.3	2.3 ± 0.2	17.3 ± 0.6	19.4 ± 0.2	13.4
Others		0.6 ± 0.2	0.6 ± 0.2	0.4 ± 0.2	1.2 ± 0.7	0.4 ± 0.1	4.9
